# Literacy transforms speech production

**DOI:** 10.3389/fpsyg.2015.01458

**Published:** 2015-09-29

**Authors:** Meredith Saletta

**Affiliations:** Department of Communication Sciences and Disorders, University of IowaIowa City, IA, USA

**Keywords:** orthography, orthographic transparency, speech production, kinematics, implicit learning

Traditionally, literacy, and speech production have been investigated separately. Studies of development demonstrate that children are able to meet the challenge of language learning across modalities, and that adults may experience difficulties in one or both modalities. Yet, it is rare to find a conceptual connection between these two processes. I argue that speaking and reading actually share important mechanisms. Specifically, orthographic characteristics of written words influence spoken as well as written language, as indicated by measures of both explicit and implicit language processing. These effects can be quantified by examining speech movement variability. An important question regarding both limb and speech motor variability is whether it is interpreted as facilitating or inhibiting the process of learning. New lines of research may explore this question by quantifying the depth of learning when stimuli are produced with greater stability or greater variability. The developmental progressions of speaking and reading also contain important parallels, which are manifest differently in individuals with varying degrees of language and reading skills. This is an important and timely issue, as it can promote theoretical accounts of language processing and respond to the clinical reality that many individuals demonstrate both spoken and written language difficulties.

## Orthographic interference

As an individual acquires literacy skills, changes occur to his/her processing of spoken, as well as written, language (e.g., Ventura et al., 2004; Ziegler et al., 2004; Alario et al., 2007; Burgos et al., 2014). This phenomenon is known in the literature as *orthographic interference;* orthography has a facilitative or disruptive effect on the perception of the spoken word. Orthographic interference affects literate individuals when they learn a new word. Learners can integrate the new word's orthographic characteristics into its mental representation, thus changing their entire perception of the word. Orthographic interference is clearly present in experiences such as the Stroop Color and Word Test (Stroop, 1935; see the review by MacLeod, 1991), in which the reader is unable to deactivate the written word's orthography. These paradigms indicate that characteristics of the written word impact the processes of both speech and reading.

The influences of orthography on perception have been well documented using behavioral paradigms. Classic studies of orthographic interference revealed that individuals who are competent speakers but illiterate, or literate only in a non-alphabetic orthography, are unable to verbally blend or segment phonemes (Morais et al., 1979; Read et al., 1986). Other early results indicate that orthography influences rhyme detection (Seidenberg and Tanenhaus, 1979), and that listeners report differences in the number of phonemes in homophones because of the presence of an additional grapheme (as in the pair “flour/flower,” in which the second spelling was often thought to have an extra phoneme; Ehri and Wilce, 1980).

Recent works examining this phenomenon have focused on reaction time (Miller and Swick, 2003; Ziegler and Muneaux, 2007) or priming effects (Damian and Bowers, 2003). Rastle et al. (2011) manipulated spelling-sound consistency in novel words during picture naming and auditory lexical decision tasks, and determined that orthographic factors influence speech production even when the speaker is not reading. Orthographic interference has also been examined via imaging studies, including measures of event-related potentials (ERP; Weber-Fox et al., 2003; Pattamadilok et al., 2009), positron emission tomography (PET; Castro-Caldas and Reis, 2003), and functional magnetic resonance imaging (fMRI; Shankweiler et al., 2008).

## Influences of orthography on implicit processing

Most of the above works focused on explicit learning. Participants were faced with a choice or required to give a response based on their conscious awareness of the stimuli. Fewer studies have investigated the influence of orthography on implicit learning. This type of processing can be quantified by measuring motor learning, which does not require conscious awareness; the participant need only produce the stimuli, not make decisions about them. Researchers may quantify individuals' articulatory stability as they speak and read aloud—a highly promising measure which provides a window into implicit learning.

Measures of articulatory stability have been used to assess implicit processing in relation to task load in children and adults with typical language development or speech and language disorders (Goffman et al., 2007; McMillan et al., 2009; Heisler et al., 2010). In these measures, kinematic parameters of movement are quantified, and the degree to which repeated movements, or productions of an utterance, converge on a single underlying template are then determined (Smith et al., 2000). These measures have been used to examine diverse phenomena in speech and language production, from the effects of altering a single phoneme (Goffman and Smith, 1999), to development and maturation (Wohlert and Smith, 1998), to stuttering and other motor speech disorders (Kleinow et al., 2001).

For the first time, this measure has been applied to individuals with differences in reading skills (Saletta et al., 2015). We indexed implicit learning by analyzing participants' segmental accuracy and articulatory stability as they learned non-words varying in modality of presentation (auditory or written) and orthographic transparency (transparent/consistent spelling vs. opaque/inconsistent spelling). Findings indicate that speech production is more accurate when non-word stimuli are read aloud than when they are simply heard and repeated. Crucially, this increase in accuracy is present even after the written text is removed. This indicates that the speakers integrated the orthographic characteristics of the non-words into their lexical representations, and supports conceptualizing reading as an interactive (rather than strictly top-down) process.

## Movement variability: Adaptive or negative?

When examining these speech production findings, a crucial point is that the interpretation of the increased stability is unclear. Traditionally, movement stability has been viewed from the perspective that greater stability is indicative of superior learning or production efficiency, and greater variability is a negative process. For instance, researchers exploring quiet stance on a forceplate considered increased sway to represent postural instability and decreased sway to indicate greater stability (Woollacott et al., 1986). Greater variability has been shown in elderly individuals who experience a slowing of online sensorimotor mechanisms, rendering them less able to modulate their sway (Fraizer and Mitra, 2008). Within the speech domain, children have been found to be more variable in their articulatory output than adults (Smith and Zelaznik, 2004), and clinical populations, such as individuals who stutter, are also generally more variable (MacPherson and Smith, 2013).

However, when investigating this effect more deeply, this interpretation is unclear. Greater movement variability may be an adaptive process which facilitates learning. In conditions of learning, such as when a child's system develops or an adult's system changes due to aging, motor variability can indicate flexibility. While perhaps counterintuitive, this has been shown in the motor control literature in several paradigms. Healthy adults and patients with Parkinson's disease may demonstrate increased sway as a strategy to enable the individual to overcome perturbations to his/her balance. In these cases, a decrease in postural sway could point toward stiffening and freezing of the degrees of freedom, reducing the individual's ability to recover from a perturbation (Chagdes et al., in revision). Studies of infants' reaching trajectories indicate that reaching is not restricted to the arm independently, but differs depending upon the body's posture, reaching from different positions and at different speeds, the freedom of the other arm, and other factors. Infants experience regression of trajectory control even after practicing reaching for several months, which indicates that early variability actually facilitates learning (Thelen and Spencer, 1998). Waddington and Adams (2003) discovered that wearing textured insoles to increase movement discrimination improved soccer players' abilities to discriminate ankle inversion, thus potentially diminishing their risk of lower limb injury. From this paradigm, Davids et al. (2003) argue that variability of motor output is essential for individuals to adapt to dynamic environments.

Viewing postural or limb motor variability as an adaptive process may be more intuitive than applying this concept to speech variability. However, it is important to note that increased variability in speech production is not always a function of a disordered system. Rather, it may actually aid developing speech and language learners in finding the optimal and dynamically changing (flexible) production patterns. We can apply this perspective to individuals' articulatory stability when speaking or reading aloud. Our previous work (Saletta et al., 2015) indicates that speech movement was more variable when reading words which were presented in the written modality with a relatively opaque spelling. Based on the motor control literature, we may conclude that participants' speech movements became more variable when they were exposed to orthography in the more challenging task because the participants were compelled to interact with the words at a deeper level. This facilitates their reorganization of their representations of the non-word and the integration of this new information.

## Influences of orthography on poor readers

The interaction between speaking and reading aloud varies across individuals with differing degrees of reading skill. Children who are acquiring reading skills atypically may fail to integrate orthographic information into their process of developing phonological representations. Speech and reading development contain important parallels. In children with typical development, the processing of spoken language follows a continuum from holistic to segmental processing. According to Nittrouer et al. (1989), children's earliest language is mediated by meaning. The earliest contrastive unit used by children is often one or a few syllables composing the word or formulaic phrase, rather than the phoneme or feature. By their second birthdays, children begin to reorganize their phonological processing from the whole word to a more segmental level (Dodd and McIntosh, 2009). Then, as toddlers mature into preschoolers, differentiation below the level of the syllable gradually emerges.

The onset of reading contributes to another reorganization, similar to that observed in spoken language. En route to achieving reading expertise, children pass through several stages. To achieve proficient reading, there is first a visual/logographic stage, during which children utilize salient graphic features to recognize the printed word (Masonheimer et al., 1984). This emergent literacy period gives way to the alphabetic stage, in which children are able to use the rules of grapheme-phoneme correspondence to decode new words (Kamhi and Catts, 2012). Proficient readers can achieve a more automatic identification of written words via visual sight word recognition (Ventura et al., 2007). Ultimately, children with reading difficulties fail to perform this reorganization effectively and efficiently. Although not every theorist supports this stage hypothesis of learning to read (e.g., Stuart and Coltheart, 1988)—indeed, specifically, there may not be a logographic stage in languages with regular orthographies (Wimmer and Hummer, 1990)—it is remarkable to consider how similarly the developmental courses of speaking and reading proceed, further supporting the interaction of these two phenomena.

This transformation is also apparent in the differences between typical and atypical adult readers (Castro-Caldas and Reis, 2003; Ziegler et al., 2003). Difficulty in acquiring literacy skills has cascading effects on neural organization. Numerous neuroimaging studies have revealed differences in visual skills (Dehaene et al., 2015) and language processing in adults with poor reading skills (e.g., Shankweiler et al., 2008). Adults with reading disabilities may use a relatively global or coarse coding rather than the fine-grained grapheme-phoneme mappings used by typical readers. This means that they may rely to a greater extent on words' visual characteristics than their phonological characteristics (Lavidor et al., 2006), and thus, that poor readers are more influenced by orthographic irregularities. In contrast, according to Bolger et al. (2008), more proficient readers are influenced to a greater degree by phonological/orthographic inconsistency. Thus, individuals with higher reading skills should be more sensitive to changes to orthographic transparency. It remains to be seen which of these conclusions receives empirical support in future studies examining implicit learning and speech production. Furthermore, these differences may be more apparent in languages with varying degrees of orthographic consistency. Serrano and Defior (2008) state that languages with greater orthographic transparency may be associated with less severe reading difficulties. Furthermore, children with reading impairment may experience greater difficulties when reading languages which are more opaque (Kamhi and Catts, 2012).

## Conclusions

Speaking and reading aloud are connected by shared mechanisms of processing and learning. Orthography influences not only reading, but speech production as well. Both reading and speaking are influenced by input, such as whether a new word is heard or read, in that reading and speaking (i.e., reading aloud) increases accuracy and stability over hearing and speaking (i.e., repeating). Speech production, from phonological encoding and articulatory planning to articulatory execution, is profoundly transformed by orthographic knowledge. Adding more auditory input does not change the production of the new word, but adding orthographic input may increase speech accuracy and cause shifts in articulatory variability. It is possible that, unlike previous interpretations of limb movement variability, speech movement variability might actually be an adaptive process which promotes depth of learning. Literate individuals can, and do, integrate orthography into a new word's representation—even without making a conscious decision to do so. All of these effects differ in speakers with varying degrees of reading proficiency. Ultimately, a word's written characteristics impact even the performance of tasks which do not involve written text. These concepts support the idea of reading and speaking as interactive processes which are mediated by differences in reading skill.

### Conflict of interest statement

The author declares that the research was conducted in the absence of any commercial or financial relationships that could be construed as a potential conflict of interest.
